# Children’s Independent Mobility and Physical Activity during the COVID-19 Pandemic: A Qualitative Study with Families

**DOI:** 10.3390/ijerph18094481

**Published:** 2021-04-23

**Authors:** Chelsea A. Pelletier, Katie Cornish, Caroline Sanders

**Affiliations:** 1 School of Health Sciences, University of Northern British Columbia, Prince George, BC V2N 4Z9, Canada; 2 Health Research Institute, University of Northern British Columbia, Prince George, BC V2N 4Z9, Canada; katie.cornish@unbc.ca; 3 School of Nursing, University of Northern British Columbia, Prince George, BC V2N 4Z9, Canada; caroline.sanders@unbc.ca

**Keywords:** physical activity, exercise, outdoor play, COVID-19, family research, interview, active transportation, rural health

## Abstract

Children’s independent mobility (CIM) is the freedom of children to move around their neighbourhood without adult supervision and is closely related to overall physical activity participation. The COVID-19 pandemic has impacted movement behaviours for children, with evidence indicating a decrease in physical activity. The aim of this study was to explore experiences of CIM and physical activity during the COVID-19 pandemic from the perspectives of children and their parents. We completed 21 family (at least one parent and one child aged 7–12) semi-structured interviews with 45 participants living in small urban and rural areas of British Columbia, Canada. Three themes were identified through a reflexive thematic analysis: (1) keeping everyone safe from COVID-19; (2) change in pattern and types of activity; (3) social impacts with family, friends, and community. Participants expressed a perceived increase in unstructured activity and a decrease in structured physical activity during the pandemic, which many parents viewed as a positive change. Parents and children indicated negative feelings due to spending less time with peers and reflected positively about spending more time with family. Parents and children expressed fear and anxiety in trying to keep their families safe from virus spread and creativity in adapting play behaviours. Findings highlight the impact of the pandemic on social friendship networks for families and a shift in activity patterns for children toward unstructured play.

## 1. Introduction

Children’s independent mobility (CIM) represents the ability for children to move independently in their neighbourhoods or communities [1,2]. Additionally known as active free play, CIM can include active travel and any indoor or outdoor unorganized activity without direct adult supervision. Children who engage in more independent mobility, such as playing outdoors and walking or cycling to school, are more likely to meet movement guidelines, report better mental health outcomes, and improved cardiovascular fitness [3,4,5]. CIM and physical activity have developmental benefits for children including improved risk assessment, decision-making skill development, increased interaction with peers, and improved self-confidence [6,7,8]. Neighbourhood environmental factors including proximity to spaces to be active (e.g., schools, parks) and parental perceptions of safety are important factors influencing outdoor physical activity and CIM [4,9,10,11,12,13]. There has been a significant decrease in CIM over the past 30 years due to changes in neighbourhood environments (e.g., urban sprawl), societal perceptions of risk, and trends toward more organized structured activities and sport participation for children [4,14,15].

The COVID-19 pandemic has impacted healthy movement behaviours for children, including physical activity and sedentary behaviour, although the pattern of these changes is inconsistent across the literature. Most studies evaluating changes in children’s health behaviours during the pandemic have identified a decrease in physical activity and increase in sedentary behaviour [16,17,18]. While some findings indicate a change in the pattern of children’s behaviour away from highly structured activities to more unstructured play [17], other studies have reported an equal percentage of parents reporting increases and decreases in outdoor physical activity in their children [19] or a perceived decrease in play in public spaces [20]. Factors associated with an increase in physical activity during the pandemic were family encouragement, parental engagement, and dog ownership [16]. Variation in changes to movement behaviours during the pandemic has been attributed to regional difference in public health policies including school, facility and park closures [21,22], neighbourhood environments such as population density and proximity to major roads [20], and family-level factors such as living in a single-family versus multi-family dwelling [16,23]. Previous qualitative work conducted in urban areas has identified parental stress, uncertainty in navigating pandemic restrictions, and reduced access to green spaces as factors negatively impacting children’s movement behaviours during COVID-19 [23]. Given the noted impact of community and neighbourhood environments on children’s movement behaviours during the pandemic [16,20], it is important to explore the experiences of families living in a variety of contexts.

In addition to a changing pattern of play, the pandemic has impacted social patterns for children who typically engage with peers at school or through physical activity. Studies have found decreased time spent with friends indoors and outdoors during the pandemic, which may be mediated by parental anxiety toward COVID-19 [19,20]. With physical distancing guidelines in place and the outdoors thought to be a lower site of transmission [24], understanding how families have experienced CIM and physical activity is important for the development of strategies and messaging to encourage healthy movement behaviours and address anxieties related to disease transmission. While previous Canadian studies have explored movement behaviours from a parental perspective [16,20,23], we are not aware of any studies that have directly included children in data generation. Capturing the qualitative experiences of children is necessary to provide nuance to our understanding of the pandemic’s impact on this group who have typically limited input in decision making, may have limited understanding of COVID-19-associated risk, and are experiencing critical developmental milestones during a period of global uncertainty [25]. The qualitative experiences of children will provide a contextual lens to complement epidemiological data [26,27] and support the development of strategies to support children during and after the pandemic.

The aim of this study was to explore experiences of CIM and physical activity during the COVID-19 pandemic from the perspective of children and their parents.

## 2. Materials and Methods

### 2.1. Study Context

Data collection and recruitment for this study occurred from September–December 2020 in northern British Columbia, Canada. Pandemic restrictions during this data collection period began with a “safe six” policy and then transitioned to restrictions of no social gatherings outside of the household on 19 November 2020 which remained in place until March of 2021 [28]. The typical school year in the region spans September–June. Because of the pandemic, in-person classes ceased in March 2020. Students had the option to return to in-person learning in June 2020 and schools opened for the new school year in early September 2020.

### 2.2. Sample and Recruitment

This analysis is part of a larger project exploring CIM in northern British Columbia using qualitative and art-based approaches. Participants included children between the ages of seven and twelve and their parents or guardians living in a northern British Columbia community (see [29] for a detailed description of the region). The age range seven to twelve yearswas chosen to be consistent with previous literature and because evidence suggests this is when independent mobility behaviours develop [30,31]. Participants were recruited through affiliated networks and organizational connections of the research team. A recruitment poster was shared through local health and child wellness organization’s social media platforms (Facebook, Twitter, and Instagram). Research ethics board approval was obtained from the University of Northern British Columbia (H20-01781). Parent participants provided informed consent for themselves and their child. Informed assent was obtained from each child participant.

### 2.3. Interview Process

A study co-ordinator met with interested participants virtually over Zoom or telephone for a pre-meeting one to two weeks prior to the interview. The pre-meeting included an overview and expectations of the study to discuss any anxieties children or parents had about joining the study. A “family” interview was considered a parent–child dyad or triad (more than one parent or child). Parents were also able to participate in the study without their children present, although none chose this option. At the interviews, basic demographic information was collected in addition to variables known to impact CIM (other children in the household, cellphone use, dog ownership, dwelling type, accessibility or learning needs) [12,22]. The interview followed a semi-structured guide with specific questions for children and parents. Where necessary, the interviewer used the child’s name to prompt a response and left longer pauses for children to respond. Interviews took between 38 min and one hour (average length: 41 min). Children were supported through the interview by allowing for flexible and accommodating timing, asking simple questions, and inviting them to come and go from the interview as they wanted.

All interviews were conducted using the Zoom videoconference platform; children and parents were given the choice of audio-only or audio and video meetings. Interview questions asked participating families about their experiences and comfort with independent and unsupervised play (e.g., “what do you think about children moving around their neighbourhood without adult supervision”); how the COVID-19 pandemic has impacted children’s independence and physical activity (e.g., “do you feel your child’s activity or play has changed during the pandemic?”; “has your play changed during COVID?”; “what type of activities do you like to do?”; “who do you like to play with? has who you play with changed during COVID”); and about access to spaces to be active during the pandemic (e.g., “where do you like to play? has where you play changed during COVID?”). Interviews were conducted by two team members. One facilitated the semi-structured interview guide and the other took field notes used to provide contextual information during analysis. Interviews were audio-recorded and transcribed verbatim by a professional transcriptionist. One child participant was not comfortable with audio recording and detailed notes of the interview were taken and analysed in lieu of a transcript.

### 2.4. Data Analysis

We chose a reflexive thematic approach to analysis as it is appropriate to conceptualize shared meaning, is iterative, allows for data immersion, and can combine the semantic and latent interpretation of the data [32,33]. Thematic analysis was conducted by three team members with backgrounds in physical activity research (CP), pediatric nursing and qualitative inquiry (CS), and mental health and well-being in rural communities (KC). Reflexivity was introduced throughout the analysis process through field notes, debriefing discussions after each interview, and discussion among team members as ideas were developed through stages of data generation and analysis. Although not necessarily recommended for a reflexive thematic analysis approach, the engagement of three researchers in analysis allowed for reflexivity through iterative discussion and refinement during theme development, and rich interpretation of latent meaning as each team member brought a different perspective and subjective experience [34,35]. Team members first familiarized themselves with the data by reading and re-reading transcripts and field notes, followed by open coding of transcripts. Initial themes were generated by each team member and then reviewed through discussion among team members to reflect on the meaning, interpretation, definition, and naming.

## 3. Results

### 3.1. Participants

Twenty-one interviews were completed with 45 participants (Table 1). Most interviews (*n* = 18) involved a parent–child dyad, three interviews included three individuals (either two parents and one child, or one parent and more than one child). Overall, 95.2% (20/21) of participating families reporting two adults living in the home and 90.5% (19/21) had other children in the home.

### 3.2. Themes

Following a reflexive thematic analysis, we developed three themes to describe the experiences and perceptions of CIM and physical activity during the COVID-19 pandemic for parents and children: (1) keeping everyone safe from COVID-19; (2) change in pattern and types of activity; and (3) social impacts with family, friends, and community (Table 2). Themes are evidenced by quotes from parents (pP) or children (cP) followed by the interview number (e.g, pP1).

#### 3.2.1. Theme 1: Keeping Everyone Safe from COVID-19

As a theme, keeping everyone safe from COVID-19 was the central concept including two sub-themes: (1) adapting to rules and guidelines; and (2) fear and anxiety in self and others. Children focused on their experiences and challenges in adapting their life, play, and daily routines to COVID-19 public health regulations and behaviours. For example, when asked what they did to stay safe while playing, cP17 indicated “we keep two meters. And we don’t go to friends’ houses.” Some children reflected on how COVID-19 impacted their play, with cP16 stating “not how much we play, but maybe what is allowed.” All children identified strategies they were using to mitigate risk while still finding opportunities to play with their peers and explained their evolving understanding of virus transmission. When asked how they stay safe while playing and how they learned to stay safe, cP4 described:

“In class we’re doing this experiment and we have to wash our hands more because we put pepper in water and when we add soap it makes the germs go away, so I’m going to wash my hands more.”

While children focused on their own experiences and fears, parents talked about adjusting to new regulations, minimizing risk, and setting boundaries:

“They [children] always play outside as opposed to inside just to kind of minimize the risk.”

(pP10)

As part of adjusting to new regulations and minimizing risk for their families, parents talked about collaborating with other parents to make decisions around COVID-19 safe rules and expectations for children when playing together:

“The moms we kind of had a conversation and said ok, well we’re going to let them start going biking, but there’s rules around COVID, right. And they didn’t go into each other’s houses and they weren’t allowed to share food, they weren’t allowed to share their bikes and stuff like that.”

(pP9)

While most families discussed a period of adjustment when new rules (COVID-19 restrictions and physical distancing) were put in place, some parents expressed difficulty in teaching their children how to follow physical distancing guidelines so they could still engage in independent mobility and physical activity, as pP11 explains:

“The most difficult thing was just trying to help them appreciate distancing issues in playing with their friends.”

Children mentioned concerns around COVID-19 disease transmission related to social activities, attending school, and their physical activity or independent mobility. When asked if they had specific fears, one child (cP17) worried about “grandma and grandpa getting sick with COVID”. Children also described sharing stories about COVID-19 between themselves resulting in anxiety:

“I do not want to get it because there’s all sorts of Coronas… one of my friends told me he had one and it was really really bad, I’m kind of worried, very worried about it.”

(cP4)

In turn, the subsequent anxieties about COVID-19 impacted children’s comfort in engaging in physical activity, as pP8 shared:

“My husband has some health issues, so [child] knows. When it [COVID-19] first came out [child] was terrified. He didn’t even want to leave the house and for a month he didn’t even go for a walk.”

Parents were anxious about their child’s play behaviour in public settings like parks, and the behaviours of other parents and children. Parents expressed feeling unsure about who they could trust (e.g., which families were following the COVID-19 regulations and who were not). Observations of other children’s behaviour in public settings impacted parent’s decisions around which children and families were safest to engage with socially or as playmates. As pP13 explains:

“Talking about kids that are down in the park that we’re not acquainted with, I don’t know the parents. So I do have some concerns about those families following the rules—are they preventing people from coming in their house and things like that.”

#### 3.2.2. Theme 2: Change in Pattern and Types of Activity

As a theme, change in pattern and types of activity reflects a perceived shift in play behaviours by parents and children and includes the subthemes: (1) increase in unstructured play; and (2) other influences on movement behaviours. Parents and children identified an increase in unstructured, imaginative, and unscheduled playtime. This included an increased reliance on CIM to stay active reflected in more time playing outside in the yard, playing unsupervised in areas of the home (e.g., the basement or garage), and required increased creativity on the part of children to occupy themselves. Parents described how their children were forced to learn how to fill their time independently since there was less reliance on structured activity and playing with peers:

“Both of them together got a lot more independent and played a lot more with each other rather than organized sports because there aren’t any, you know they had to kind of self-entertain.”

(pP20)

Children described similar changes in their play routines, including playing outside more, as cP18 stated” I’ve been playing outside a lot more because he [dad] made me, but also because I choose to.” Other children indicated it was not necessarily a bad change, but one forcing them to try new things and spend more time outside. cP14 describes these changes to their activity patterns:

“Different I would say. Well, I don’t think we’re doing less cause like we were doing more like the same amount of activity but like outside, but just like different, like more of a variety of things and now we’re just biking to school, we’re going like to the park and bike park pretty much. And we’ll be skiing soon.”

Some children reflected on how they were forced to adapt their activity due to restrictions and closures of indoor public places, resulting in more outdoor unstructured play:

“I would have been going to school and play at like just the pool and the museum and stuff, but now I’ve been playing more at parks than the pool.”

(cP10)

Some children described finding new activities to engage in, which impacted their social friendships:

“I got a lot more into mountain biking and I met with, like a smaller friend group that I got a bit closer to than I would’ve if it [the pandemic] didn’t start because I wouldn’t be forced to hang out with a smaller group of people.”

(cP9)

Many parents indicated the decrease in highly structured play and extra-curricular activities was a positive change and COVID-19 associated closures provided an opportunity to reflect on their previous busy daily routines. In reviewing prior activity and organized sport participation, pP19 found a sense of relief and reduced pressure:

“Swimming started again, but like it’s a limited schedule. So instead of swimming five days a week, she’s swimming two days a week and that’s been great...it just feels like we have more free time. I feel like both of them [children] had too much to do, and in March it was kind of a relief to do nothing.”

Associated with the decrease in structured extra-curricular activities, parents noted more time for family activities, as pP1 explained:

“I would say this year is different than other years in terms of less organized activities that both my kids are in. For me it’s way less stress, we can always have dinner together because young children’s activities are always between like 4 and 7 so someone’s always got to be picking up, dropping off, all that kind of thing.”

In contrast to the positive feelings and stress relief expressed by parents, some children were disappointed that organized sports and activities were cancelled or significantly modified, cP1 explains:

“This is the first year that I get to play like school sports (team sports), but I don’t know if I’ll be able to play some of them because of the pandemic.”

Parents and children explained their pattern of activity changed over the course of the pandemic due to seasonal changes and school opening and closing, as described by pP3:

“This spring the kids were home out of school, so we did more activities outside and more outdoor activities with friends than we normally would’ve done in a normal spring, because this was a way that we felt more comfortable for the kids to get together during COVID and not be unsafe.”

In addition to the influence of seasons and the school year, there were changes in access to public spaces commonly used by children for physical activity, impacting the pattern and types of activity options. As pP4 explains, these closures limited opportunities for independent mobility and physical activity:

“Our parks around town were closed up for a while, like we were told not to go on them. The pool is shut down, the skating rink is shut down, the climbing wall has been shut down, basically the only thing open right now in town is playgrounds for kids.”

Children similarly described a loss in play opportunities based on the closure of public spaces and how new rules limited their ability to roam and explore:

“Sometimes we don’t get to go anywhere at school anymore, we’ve got certain areas we get to go to, like we have to stay at the field all week and we can’t to the other places [other areas to play on school grounds].”

(cP4)

#### 3.2.3. Theme 3: Social Impacts with Family, Friends, and Community

The theme of social impacts with family, friends, and community describes the role of the COVID-19 pandemic on social opportunities and how these changes altered CIM and physical activity engagement. Identified subthemes include: (1) spending more time with family; and (2) fewer opportunities for peer socialization. In response to physical distancing guidelines, participants described a loss of social opportunities. The loss of social freedom contributed to a decrease in independent mobility in children. Because children typically engage socially through movement, the loss of social opportunities was both a result of having fewer opportunities to be active and contributed to different patterns of activity.

For children, the loss in social engagement was related to a decreased participation with their peers in organized and unorganized physical activity. When asked about friendship networks in their neighbourhood, cP17 expressed “we do [have friends in neighbourhood], but since COVID we can’t play that much.” The change in social opportunities led to a change in activity options. In some cases, children adapted and engaged in CIM without their peers, as cP06 expressed:

“Definitely. Probably like in the summer I probably would’ve been like on my skateboard going in skate spots with my friends, um, but I couldn’t hang out with a lot of my friends. And so I didn’t have the same interaction and so I just go alone. I also couldn’t have any friends over a lot of the time.”

Parents similarly reflected on how the reduction in social activities impacted independent mobility and physical activity for their children. There were also concerns raised by parents about how this would influence mental health and social development. For example, pP9 explained how COVID-19 restrictions impacted their child:

“He’s very social, he’s an extrovert, and it took a toll on him. By the time we were at the place in our community where we were able to kind of start getting back into activities and things like that, it couldn’t have come at a better time for him because he was starting to go nuts.”

To combat the reduced social activity, parents explained how it was important to allow their children to engage in independent mobility and physical activity with peers to support mental health.. In responding about changes in their child’s play, pP16 mentioned:

“Not a lot [of change], the kids play a bit further apart, but we made a street bubble. The kids needed it for their mental health.”

Despite changing opportunities to engage with peers, parents and children expressed their children’s activity did not really decrease, but who they played with changed. In particular, children were playing more with siblings, as pP3 described “they’re still playing, so that’s good, it’s just with the sibling group rather than friends.” Similarly, cP14 explained “we had less play dates, like gatherings.”

Parents and children both expressed that engaging less with peer groups meant more time with family. As cP17 mentioned “I’m playing more with my sister, and I play on my own sometimes.” Parents in particular expressed positive feelings related to spending more time with family and children engaging more with siblings:

“During COVID we weren’t seeing friends as much and it actually forced her to get along with her sister better which was good. I mean at first it was terrible, like they fought so much, but after a while it’s like they had to fight it out and then they start to get along playing together.”

(pP15)

## 4. Discussion

Based on the perspectives of children and parents, findings from this study indicate a perceived increase in unstructured play during the COVID-19 pandemic and impacts on social opportunities for families. We present a novel qualitative contribution to existing quantitative data exploring COVID-19-related changes in children’s movement behaviours, include the perceptions of children regarding their pandemic experiences, and provide data from families in non-urban contexts. Online self-reported surveys have identified a general decrease in physical activity during the pandemic, including a reduction in the number of children meeting physical activity guidelines and a specific reduction in some domains of physical activity including outside play and sport [16], even after school and sports facilities re-opened [21]. Findings from our current work support these overall trends and provide a nuanced description of how families are coping with COVID-19 restrictions, reflecting daily anxieties related to disease transmission and ongoing adaptations to play routines to maintain friendship networks.

Parents identified a perceived increase in unstructured activities and recognition of doing too much pre-pandemic. During the pandemic, families identified they could still be active without as many structured activities, increasing independent mobility and unstructured play. Other work has similarly noted the reclaiming of streets and public spaces for kids as they explored and engaged with outdoor creative play and found strategies to connect with one another while adhering to facility closures and physical distancing guidelines [36]. This finding is noteworthy considering the general decrease in CIM among children in westernized countries since at least the 1990s, coinciding with an increase in structured play and extracurricular activities [11,14,15]. Many of these pandemic-induced changes were seen as positive by parents as they expressed reduced stress related to busy daily schedules and increased family bonding. While some children expressed neutral feelings toward their new play routines or a lack of change, others expressed disappointment in not being able to play organised sports, access facilities, or play with their friends. As countries begin transitioning out of the pandemic, it is important to find ways to foster creative unstructured play for children, support families to maintain positive increases in CIM, and mobilize strategies to enable children to remain active with their friends while protecting against virus spread.

Similar to other work [19], our findings identify the social impacts of COVID-19 including spending more time with family at the expense of interactions with peers. Families described fewer or no playdates, maintaining physical distancing, and not being allowed in other people’s homes, resulting in a loss or reduction of their friendship networks. To support their child’s mental health and keep everyone safe, parents described their decision process around who to include in their limited social circles. At times, there was an opportunity to form new community bubbles with neighbours to ensure active social engagement and support mental health. Similar reflections have been identified among families in Australia, including being stressed and missing out on important social activities as well as disruption in routines and activities [37]. Children may be especially vulnerable during the COVID-19 pandemic as they are in critical periods of development and have limited control over their own circumstances or decision-making, impacting mental health [25,38]. CIM and physical activity are associated with the positive development and well-being of children and are often one of the main ways children and families form peer networks [9,15,39]. As a health promotion strategy, supporting independent free play and physical activity may ameliorate the potential negative social and mental health impacts associated with the pandemic in children.

The region of northern BC is characterised by rural and small urban settings [29,40]. This was reflected in demographic characteristics as all participants resided in single-family homes and in communities with a population of fewer than 75,000 people, with many living in communities with a population of fewer than 5000 people. Prior studies have identified families living in areas with a lower population density and greater distance from major roads have been more likely to sustain positive movement behaviours during pandemic restrictions [20]. Our findings support this general trend and further suggest children in rural communities may have more opportunities to engage in CIM during the pandemic or be more comfortable with outdoor play, making the transition away from structured activities easier. On the other hand, children living in rural communities may experience unique barriers to CIM and physical activity related to weather, wildlife, and access to destinations [41,42,43], highlighting the need for context-specific supports and research. Our prior work with adults living in this region has identified the close association between community engagement, social participation, and physical activity [44], a finding consistent with families in the present study based on the close relationship between CIM, physical activity, and social participation in their communities. Although a comparison between regions was out of the scope of this project, social engagement is an important facilitator of physical activity behaviour for children, and may be an important mediating factor in COVID-19-associated changes in movement behaviours, particularly in rural areas [15,39,45]. Overall, our findings suggest families living in rural and urban areas may be experiencing the pandemic differently, resulting in divergent impacts on movement behaviours, although a more robust comparison is needed to confirm this finding.

### Limitations

Our sample has almost entirely women parental participants, with only one man. Since parental perceptions of risk can vary based on gender [12], our findings should be considered in light of this limitation. It remains important to consider gender influences on children’s movement behaviours in terms of the gender of the child and potentially of the parent as well. Although open to people living across the region, participants were recruited for this project to discuss their perceptions of CIM in the region of northern British Columbia. As a result, our sample is likely biased toward people who have a relatively high interest in physical activity and CIM and may have been more likely to encourage and value independent play during pandemic restrictions. Our demographic questionnaire did not include direct measures of socio-economic status instead capturing indirect measures like the type of home and cellphone access. Due to this, we cannot draw conclusions as to the socio-economic status of the final sample.

## 5. Conclusions

As a result of the COVID-19 pandemic, families expressed a shift toward unstructured play, a loss of social opportunities, and anxieties related to adapting to safety protocols. Although families articulated challenges and difficulties during the pandemic, there were positive changes identified related to increased freedom and flexibility from a reduction in overly scheduled activities, leading to increased CIM and opportunities for family connection. Longitudinal research is needed to capture how families are coping and adapting to the evolving pandemic and to understand if and how positive changes to movement behaviours and daily routines can be maintained post-pandemic. Recommendations for public health policy include the need to: support the mental health of children related to understanding and coping with virus anxieties; develop child-appropriate messaging of how to play safely; and implement health promotion strategies and COVID-19 public health policy that considers the benefits of independent mobility and physical activity for children.

## Figures and Tables

**Table 1:** Participant demographic characteristics.

Characteristic	Household (*n* = 21)	Parent (*n* = 22)	Child (*n* = 23)
Age (mean, range)	-	42.4, 31–57	9.6, 8–12
Gender (n, %)			
Women/Girl	-	21, 95.4%	9, 39.1%
Man/Boy	-	1, 4.5%	14, 60.9%
Community population (n, %)			
<5000	5, 23.8%	-	-
10,000–20,000	4, 19.0%	-	-
20,000–30,000	1, 4.8%	-	-
30,000–75,000	11, 52.4%	-	-
Dog ownership (n, % yes)	12, 57.1%	-	-
Dwelling type (% single-family house)	21, 100%	-	-
Learning or accessibility needs in the home	5, 23.8%	-	-
Cellphone with data	-	-	2, 8.7%
Cellphone without data (WiFi connection only)	-	-	6, 26%

**Table 2:** Themes and sub-themes identified through reflective thematic analysis of interview data.

Theme	Sub-Theme	Codes
Keeping everyone safe from COVID-19	Adapting to rules and guidelines	Unpredictable Lost playtime to accommodate safety rules and sanitation protocols
	Fear and anxiety in self and others	Judgement from other parents Worried about if others are following the rules Keeping “family” safe by restricting play and activities
Change in pattern and types of activity	Increase in unstructured play	Closed structures and places to be active Less supervision More time outside Reduced opportunities for structured activity
	Other influences on movement behaviours	School year Different weather and seasons Changing restrictions
Social impacts with families, friends, and community	Spending more time with family	Learning how to play with siblings Positive change
	Fewer opportunities for peer socialization	Important for kids and parents School closure Creative solutions and adaptations

## Data Availability

The data are not publicly available due to confidentiality concerns.
